# Influence of Processing Conditions on the Generation of Surface Defects in a Heat-and-Cool Hybrid Injection Molding Technique for Carbon Fiber-Reinforced Thermoplastic Sheets and Development of a Suitable Mold Heated by Far-Infrared Radiation

**DOI:** 10.3390/polym15224437

**Published:** 2023-11-16

**Authors:** Yasuhiko Murata, Ryunosuke Machiya, Takuma Komori

**Affiliations:** 1 Department of Mechanical Engineering, Faculty of Fundamental Engineering, Nippon Institute of Technology, 4-1 Gakuendai, Miyashiro-machi, Minamisaitama-gun, Saitama 345-8501, Japan; 2 Mechanical Systems Engineering Major, Graduate School Nippon Institute of Technology, 4-1 Gakuendai, Miyashiro-machi, Minamisaitama-gun, Saitama 345-8501, Japantk-komori@enplas.com (T.K.)

**Keywords:** carbon fiber-reinforced thermoplastic sheet, heat-and-cool hybrid injection molding, far-radiation heater, surface defect, temperature measurement

## Abstract

Recently, hybrid injection molding—a type of overmolding technology in which a short carbon fiber-reinforced thermoplastic is injection molded over a compression-molded carbon fiber-reinforced thermoplastic (CFRTP) sheet—has been introduced. A heat-and-cool hybrid injection molding technique has also been introduced for practical use. The technique yields high-quality molded products. This is achieved through the heating of the mold cavity surface to a temperature higher than the melting point of the base polymer impregnated into the carbon fiber fabric. However, few experimental analyses of the molding phenomena in heat-and-cool hybrid injection molding have been reported. In particular, the effect of the processing conditions on the transfer of the mold cavity surface shape to the CFRTP sheet has not been clarified in detail. Therefore, it has been impossible to take extensive measures when defects are generated in molded products. In this study, a mold is designed and fabricated for use with far-infrared radiation heating, a variotherm technology that is suitable for the experimental analysis of the heat-and-cool hybrid injection molding phenomenon. In particular, a mold is designed and fabricated to continuously perform the following three processes using only an injection molding machine: (1) the radiation heating of both the CFRTP sheet and the mold cavity surface using a far-infrared radiation heater, (2) the compression molding of the CFRTP sheet, and (3) the injection molding of the melt. The effects of the heating conditions of the mold and the injection molding process conditions on the appearance characteristics of the molded products are clarified using this mold and a far-infrared radiation heater.

## 1. Introduction

Carbon fiber-reinforced plastics (CFRPs), in which carbon fibers [1,2] are used as reinforcement materials, are lightweight and have excellent strength. CFRPs are used in a wide range of products, from fishing rods to structural parts of airplanes and vehicles [3,4,5]. The prepreg structure, or the fabric woven from a continuous fiber reinforced with thermosetting resin, has been the mainstream in CFRP. A long period is required for the curing reaction, resulting in long manufacturing times and other problems, making them unsuitable for mass production [6]. However, the emergence of carbon fiber-reinforced thermoplastics (CFRTPs) reinforced with short fibers with a length of several hundred micrometers to several millimeters and the application of injection molding techniques to CFRTPs have brought about a higher degree of freedom in shape, enabling the mass manufacturing of products with complex three-dimensional shapes. The problem with short fibers is their reinforcing effect, which is inferior to that of continuous fibers, which, in turn, results in the low mechanical strength of products [7]. In recent years, sheets of continuous fibers reinforced with thermoplastics have been developed, and techniques utilizing the features of both continuous and short fibers have been proposed to produce complex three-dimensional molded products with high specific strength in a short molding cycle time. Specifically, hybrid injection molding, a type of overmolding technique, has been introduced in practical use, in which a CFRTP sheet with a woven structure is first compression molded while being softened by radiation heating, and then a short carbon fiber-reinforced thermoplastic is injected over the primary molded product to produce molded products with complex shapes, such as ribs for reinforcement effects [8,9,10,11,12]. With this molding technique, however, the CFRTP sheet comes into contact with the low-temperature surface of the mold cavity during compression molding and solidifies. As a result, the transferability of the shape of the cavity surface to the molded products decreases. It has, therefore, been difficult to use a hybrid injection molding technique for the manufacturing of defect-free molded products. To address this issue, heat-and-cool hybrid injection molding using the variotherm technique has been applied. This hybrid injection molding technique involves heating the mold cavity surface to a temperature higher than the melting point of the base polymer of the CFRTP sheet [13,14].

Regarding the hybrid injection molding technique without heating and cooling, a number of studies have been conducted in a wide range of areas, from sheet materials to injection-molded polymers [15]. Among them, hybrid injection-molded products are widely studied regarding the molded parts’ strength, mainly because they are used as lightweight structural parts that do not require assembly. One such study focused on the effect of the injection molding process conditions on the adhesive mechanism and adhesive quality between fiber-reinforced thermoplastic sheets and long fiber-reinforced thermoplastics [16]. The relationship between the preheating time of the thermosetting resin sheet and injection molding process conditions, such as the temperature and injection rate of the injection-molded polymer, and the adhesive strength has been studied using measurements of the melt pressure inside the cavity and mold temperature [17,18]. It has been clarified that the preheating time has a significant effect on the adhesive strength, with higher cavity pressure and mold temperature conditions resulting in higher adhesive strength. Similar studies to the above have been conducted for CFRTP sheets using measurements of the melt pressure and temperature in the cavity [19]. It has been clarified that as the temperature of the injection-molded polymer increases and the injection rate increases, the interface temperature increases, resulting in an increase in adhesive strength. Moreover, an increase in preheating time causes roughness on the sheet surface, resulting in an increase in adhesive strength due to the anchor effect. Studies are also being conducted on pretreatment methods for sheet surfaces to increase adhesive strength. For example, the improvement in the adhesive strength in the rib part has been attempted by cutting the continuous fibers near the surface of the CFRTP sheet shorter and entangling these fibers with the injection-molded polymer at the base of the rib [20]. Furthermore, the effects of the linear expansion coefficient and the bending modulus of the CFRTP sheet and injection-molded polymer on the warpage of molded products have been examined [21]. It has been clarified that greater warpage is generated in the molded products in injection-molded polymers with higher linear expansion coefficients and in injection-molded polymers with a lower bending modulus. In addition, a study has been conducted on the influence of the environmental temperature on the molded product’s strength [22], as well as a study on the development of FEM simulation methods to assist in the design of molded products [23].

On the other hand, few studies have been conducted on heat-and-cool hybrid injection molding. Overmolding molds that can be heated by applying rapid heat cycle molding (RHCM), one of the variotherm techniques, have been fabricated to study the effect of mold heating on the surface quality of molded products [24]. It has been clarified that heating to 150 °C lowers the surface roughness Ra of CFRTP sheets from 0.43 μm to 0.06 μm, compared with conventional molding at a constant temperature of 85 °C. However, the effects of the mold heating temperature and injection molding process conditions on the surface properties and surface defects of CFRTP sheets in heat-and-cool hybrid injection molding have not been studied over a wide range of conditions. For this reason, it has been impossible to take extensive measures when the dimensional accuracy of the molded products is low, the surface properties are poor, or molding defects such as gas burning are generated.

There are several variotherm techniques for the heating and cooling of a mold, such as circulating a heating medium and a cooling medium alternately through the mold [25,26], using an electric heater [27,28], or using an electromagnetic induction-heating system [29,30,31,32,33,34,35], which have been applied in practice. A method of heating the mold surface using infrared radiation has also been proposed [36]. However, infrared radiation heating is rarely used in the manufacturing process. This is because the heating rate is lower and the molding cycle time is longer than that of the variotherm techniques described above because of the large energy losses due to reflection on metal surfaces with relatively low surface roughness, such as mold surfaces. However, compared with the above variotherm techniques, infrared radiation is used in research applications where the molding cycle time is not an issue because it is easier to heat mold surfaces where nano- and micro-shapes are processed or where surface defects are generated from outside the mold. Some of the authors of this paper have proposed a heating and cooling injection mold that can be easily heated using a far-infrared radiation heater and a three-dimensional cavity plate. The effects of heating the mold cavity surface on the appearance of molded products composed of fiber-reinforced thermoplastics have been examined using this mold [37,38]. Since an infrared radiation heater is often used to heat the sheets in overmolding, we speculated as to whether it would be possible to heat both the sheet and the mold surface simultaneously using only one far-infrared radiation heater.

In this study, the above molds [38] are used as the basis, and a mold is designed and fabricated to continuously perform the following three processes using only an injection molding machine: (1) the radiation heating of both the CFRTP sheet and the mold cavity surface using a far-infrared radiation heater, (2) the compression molding of the CFRTP sheet, and (3) the injection molding of the melt. The effects of the heating temperature conditions of the mold, the injection molding process conditions, the number of CFRTP sheet layers, and the compression ratio of the sheet on the appearance characteristics of the molded products are examined using this mold and a far-infrared radiation heater.

## 2. Experimental Methods

### 2.1. Heat-and-Cool Hybrid Injection Molding Technique

**Figure 1:**
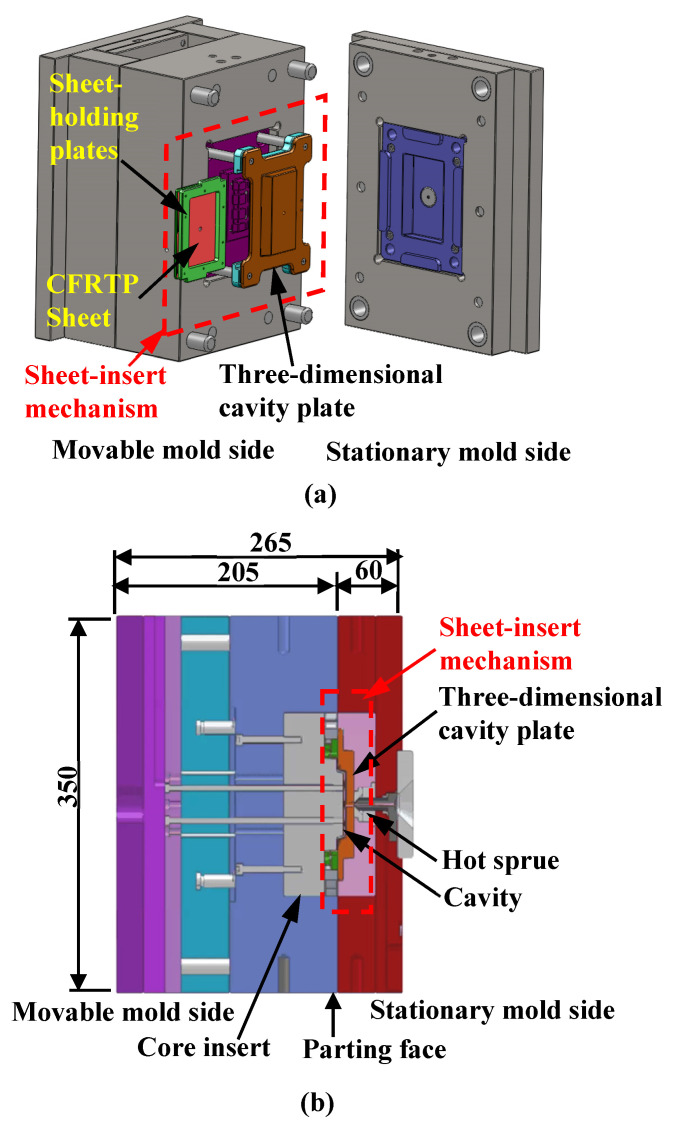
Basic structure of heat-and-cool hybrid injection mold (unit: mm): (**a**) 3D model of the mold; (**b**) cross-sectional view of the mold.

Figure 1 shows the basic structure of the mold designed and fabricated in this study, which is applied to the heat-and-cooling hybrid injection molding technique using far-infrared radiation heaters. A sheet-insert mechanism, having a three-dimensional cavity plate that is heated using a far-infrared radiation heater, is mounted on the parting face of the mold. A CFRTP sheet held between the sheet-holding plates is inserted into the sheet-insert mechanism. The mold has a three-plate structure; namely, the sheet-insert mechanism is separated from the movable mold side by the elastic force of the springs when the mold is open, as shown in Figure 1a. To prevent a sprue from interfering with the release of the molded product from the mold, a hot sprue (SK-GVII; Ju-OH Inc., Hiratsuka, Japan) is mounted on the stationary mold side. A sprue is therefore not molded. A core insert for injection molding ribs is incorporated into the movable mold side.

**Figure 2:**
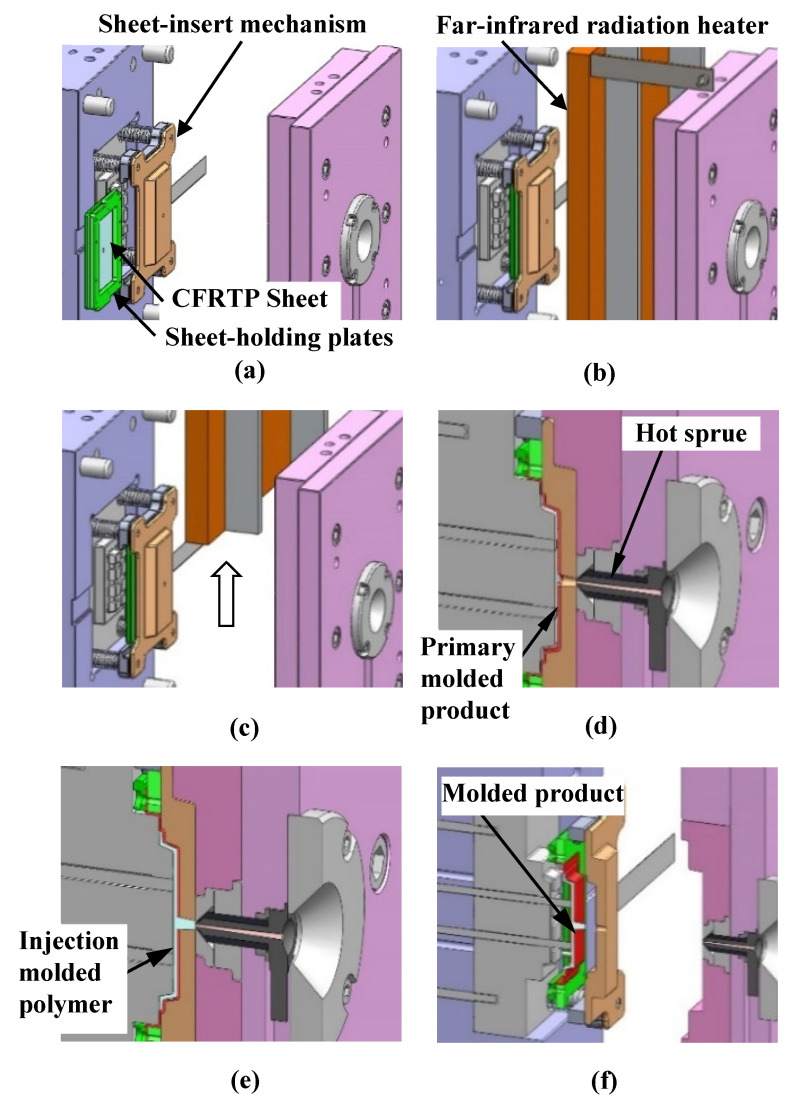
Heat-and-cool hybrid injection molding of CFRTP sheets using a far-infrared radiation heater: (**a**) sheet-insertion process; (**b**) heating process; (**c**) heater rise; (**d**) compression-molding process; (**e**) injection molding process; (**f**) mold release process.

Figure 2 shows the proposed heat-and-cool hybrid injection molding process using a far-infrared radiation heater. The CFRTP sheet is fixed by being sandwiched between the holding plates and inserted into the sheet-insert mechanism, as shown in Figure 2. The far-infrared radiation heater mounted on the upper part of the mold is lowered to the position facing the three-dimensional cavity plate to heat both the three-dimensional cavity plate and the CFRTP sheet simultaneously, as shown in Figure 2b. The heater is lifted to the original position when the three-dimensional cavity plate and the sheet are heated to a given temperature, as shown in Figure 2c. Then, the mold is clamped. With this mold clamping by the injection molding machine, the CFRTP sheet is sandwiched between the three-dimensional cavity plate and the core insert, compressed, and deformed to form the primary molded product, as shown in Figure 2d. Subsequently, the melt is injected into the cavity between the CFRTP sheet and the core insert and then fused to the surface of the sheet, as shown in Figure 2e. The three-dimensional cavity plate starts cooling when it comes into contact with the low-temperature mold base during mold clamping. The mold is opened after the completion of cooling, and the sheet-holding plates supporting the molded product are removed from the gap between the three-dimensional cavity plate and the core insert, as shown in Figure 2f. Finally, the molded product is released from the sheet-holding plates and manually trimmed to remove unnecessary portions using a blade.

**Figure 3:**
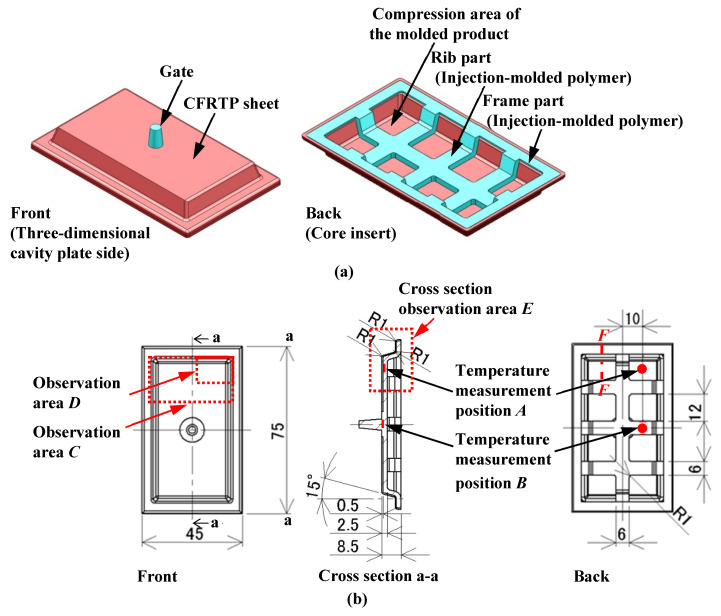
Shape and dimensions of the molded product, temperature measurement point, observation area (unit: mm): (**a**) shape; (**b**) dimensions.

Figure 3 shows the shape and dimensions of the molded product obtained by the above process. The molded product has a shallow box shape. This paper aims to test the effect of injection molding process conditions on the surface properties of overmolded products. Therefore, the strength of the molded product is not considered. The compression-molded CFRTP sheet, indicated in pink, is located on the front surface of the molded product, while the injection-molded lattice-like rib and the square frame arranged to surround the rib are indicated in blue and located on the back surface. The gate is located at the center of the molded product. The melt flows from the gate to the rib on the back surface of the CFRTP sheet through the circular hole created at the center of the sheet. The reason for machining a circular hole in the CFRTP sheet and designing a structure in which the melt flows to the back side of the sheet is as follows. A structure in which the mold is rotated 180° and the ribs are positioned on the stationary mold side is considered. However, in this case, because the product is molded on the stationary mold side, the molded product ejection mechanism of the injection molding machine cannot be used, and a new ejection mechanism must be installed on the stationary mold side, further complicating the mold structure.

### 2.2. Design and Fabrication of Mold

**Figure 4:**
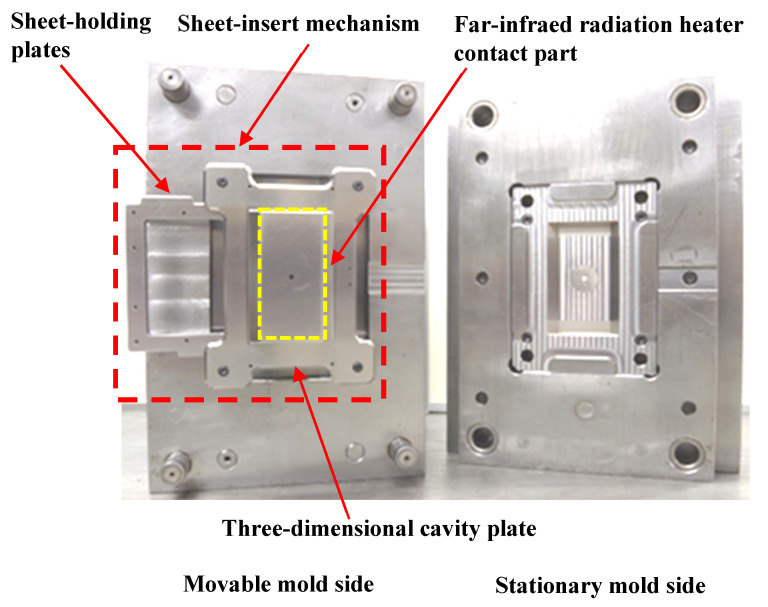
Appearance of heat-and-cool hybrid injection mold.

**Figure 5:**
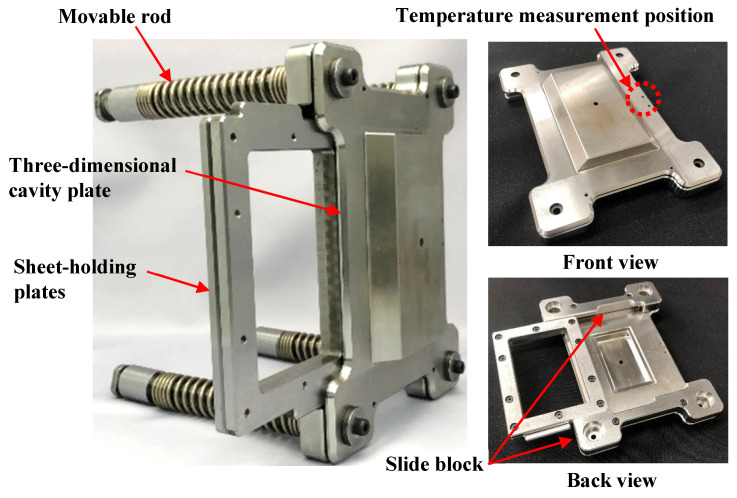
Appearance of sheet-insert mechanism.

Figure 4 shows the appearance of the heat-and-cool hybrid injection mold designed and fabricated in this study. Figure 5 shows the appearance of the sheet-insert mechanism. The sheet-insert mechanism is mounted on the movable mold side using four movable rods. Springs are attached to the movable rods, as shown in Figure 5a, such that they are sandwiched between the three-dimensional cavity plate and the core insert on the movable mold side. The three-dimensional cavity plate, therefore, is in contact with the core insert on the movable mold side when the mold is clamped and separated from the core insert owing to the elastic force of the springs when the mold is open. The sheet-insert mechanism consists of the three-dimensional cavity plate, two slide blocks, and two sheet-holding plates, as shown in Figure 5b. The sheet-holding plates holding the CFRTP sheet are inserted into the rail part, consisting of the three-dimensional cavity plate and slide blocks, as shown in Figure 2a. The periphery of the CFRTP sheet is held between the two sheet-holding plates and fixed with screws. Removal grooves are provided on the upper and lower parts of the slide blocks to allow the sheet-holding plates to be removed from the slide blocks during demolding. The material of the three-dimensional cavity plate is alloy tool steel (SKD11: JIS standard), and the cavity surface where the CFRTP sheet is in contact is mirror polished to Ra 0.105 μm.

**Figure 6:**
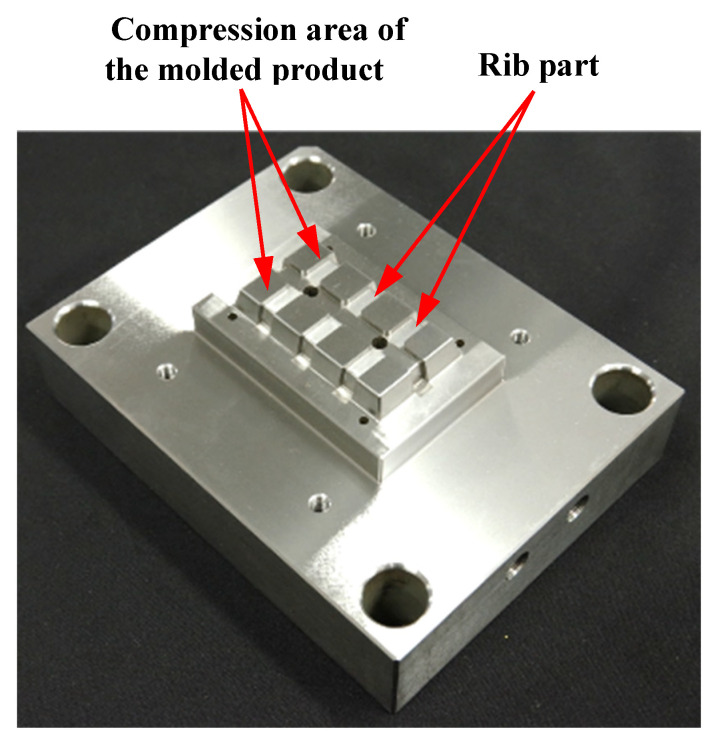
Appearance of core insert incorporated into movable mold side.

Figure 6 shows the appearance of the core insert incorporated into the movable mold side. The core insert on the movable mold side has a protruding face that presses the CFRTP sheet onto the surface of the three-dimensional cavity plate for compression molding (hereinafter referred to as the “compressed area of the molded product”) and a rib part through which the injection-molded polymer flows. It is assumed that most of the compressed base polymer of the CFRTP sheet flows into the ribs and the frame part during compression molding.

### 2.3. Experimental Method

**Figure 7:**
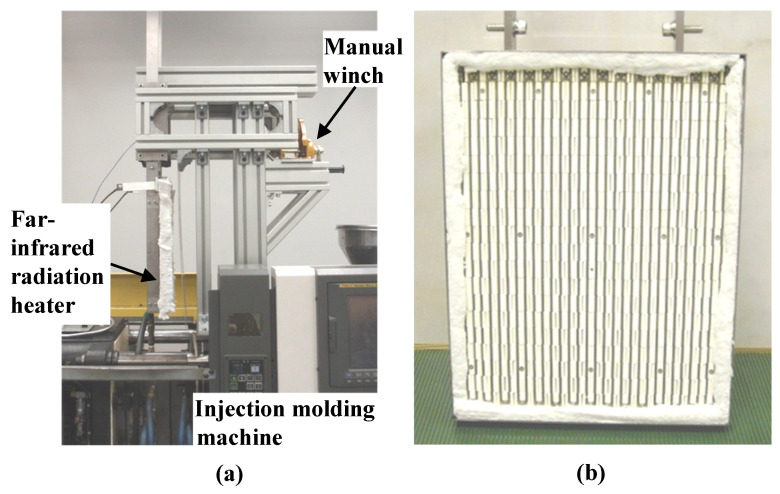
Appearance of (**a**) far-infrared radiation heater-lifting device and (**b**) far-infrared radiation heater.

Figure 7 shows the appearance of the heater-lifting device used for the lowering and lifting of the far-infrared radiation heater into and out of the mold and the far-infrared radiation heater used to heat the sheet-insert mechanism. The far-infrared radiation heater is a radiant pad heater with metal heating wires embedded in ceramics (PD3040; YAC DENKO Co., Ltd., Tokyo, Japan; 425 mm × 325 mm × 30 mm, infrared wavelength range: 3.0–7.0 μm). A manual winch designed and fabricated in a previous study [38] is used as the heater-lifting device. The injection molding machine used in the experiment is the ROBOSHOTS-2000i50A (Fanuc Corporation, Yamanashi, Japan; maximum mold-clamping force, 500 kN). A mold temperature controller (TYPE TA-32; STOLZ Co., Ltd., Murayama, Japan) using hot water is used to control the temperature of the mold base. The CFRTP sheet used is a Pyrofil sheet (TR6110; Mitsubishi Chemical Corporation, Tokyo, Japan; carbon fiber tow of 6K; fiber volume content of 60%; plain weave of 0.4 mm thickness), the base polymer of which is polymethylmethacrylate (PMMA). The injection-molded polymer is PMMA (Acrypet VH001; Mitsubishi Chemical Corporation).

**Figure 8:**
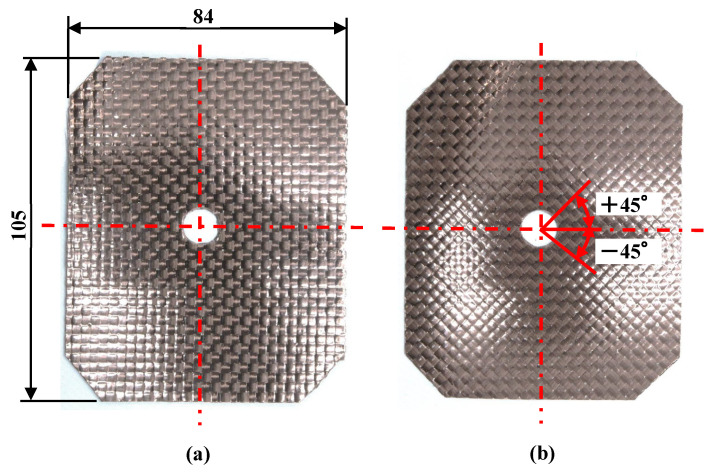
Appearance of CFRTP sheet and fiber tow orientation (unit: mm): (**a**) 0°/90°; (**b**) +45°/−45°.

**Figure 9:**
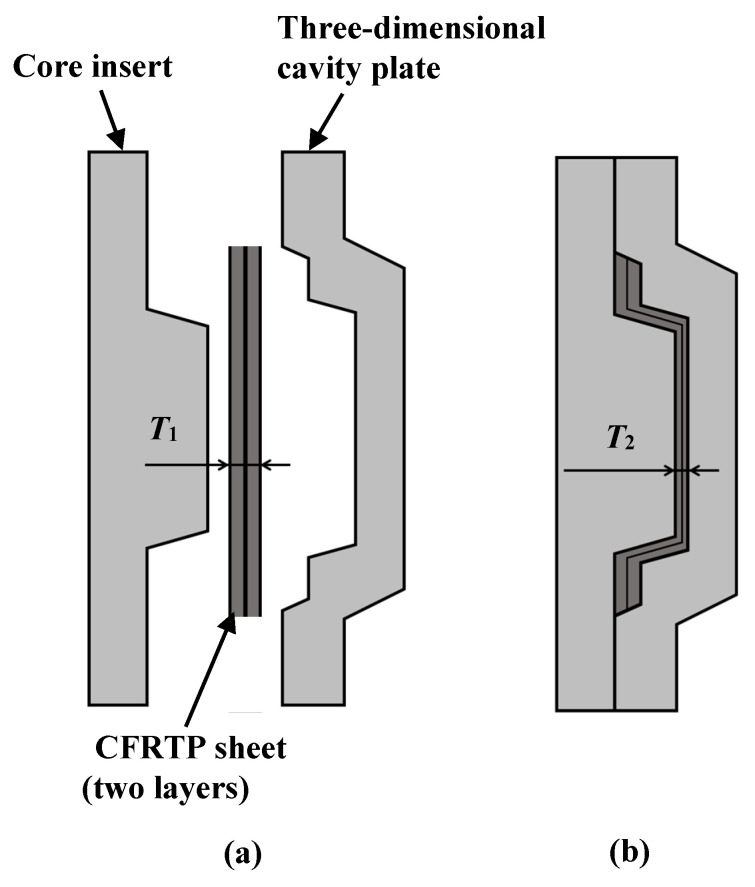
Compression ratio of CFRTP sheets: (**a**) before compression; (**b**) after compression.

Table 1 shows the molding process conditions used. In this study, injection molding process conditions such as the heating cylinder temperature, mold base temperature (excluding the three-dimensional cavity plate), hot sprue temperature, injection rate, holding pressure, and pressure holding period are set to constant values. First, the three-dimensional cavity plate is heated to a predetermined temperature from 50 °C, the set temperature of the mold base by the mold temperature controller, by a far-infrared radiation heater that is heated to 600 °C. The far-infrared radiation heater is in contact with the three-dimensional cavity plate surface as denoted in Figure 4) (distance between heater and three-dimensional plate: 0 mm). Therefore, in addition to the far-radiation heating effect, a heating effect by heat transfer from the heater to the plate is expected. Then, the CFRTP sheet is compression molded using the mold-clamping device of the injection molding machine. The pressing force during compression molding is 500 kN, which is the maximum mold-clamping force of the injection molding machine. The time from the start of the lifting of the heater from the three-dimensional cavity plate to the completion of CFRTP sheet compression is variable due to the use of the manual winch. This time is in the range of 10 s to 15 s. The melt is injected after a certain period of time. Heat in the three-dimensional cavity plate is transferred to the mold base, which is set at 50 °C from the moment that the plate comes into contact with the mold base and is cooled to 50 °C together with the molded product. The molded product is released from the mold after being cooled to 50 °C. The effects of the plate temperature on the appearance of the molded products are examined in this study by heating the three-dimensional cavity plate to four different temperatures: 180, 200, 240, and 280 °C. The effects of the elapsed time Δ*t* between the completion of the compression molding of the CFRTP sheet and the start of melt injection on the appearance of molded products are also examined by setting Δ*t* to four different values: 0, 5, 10, and 15 s. The products are molded under two different fiber tow orientations: 0°/90° and +45°/−45°, as shown in Figure 8, to examine the effects of the fiber tow orientation on the molded products. The effect of the number of CFRTP sheet layers on the molded products is also examined, specifically in cases with one and two CFRTP sheet layers. The compression ratio *α* during compression molding of the CFRTP sheets is determined as *α*% = (*T*_1_ − *T*_2_)/*T*_1_ × 100, where the total thickness of the CFRTP sheet and the cavity gap are defined as *T*_1_ mm and *T*_2_ mm, respectively, as shown in Figure 9. The mold is adjusted so that *α* remains constant even when the number of CFRTP sheet layers is changed. The effects of changes in *α* on the molded products’ appearance are then examined for each number of sheet layers. Five products are molded under each condition.

One of the objectives of this study is to examine the effects of the heating conditions of the three-dimensional cavity plate on the appearance of the molded products. For this purpose, the temperature of the three-dimensional cavity plate and that of the CFRTP sheet are measured. The temperature of the three-dimensional cavity plate is measured by embedding a chromel–alumel sheath thermocouple that is 0.5 mm in diameter (Raytherm Co., Ltd., Tokyo, Japan, Brentwood, UK) into the position on the side of the plate, as shown in Figure 5. The temperature of the CFRTP sheet is measured by attaching a temperature sensor for micro-surfaces, ST-56 (RKC INSTRUMENT INC., Tokyo, Japan), a chromel–alumel bare wire thermocouple with a strand diameter of 0.126 mm, to the CFRTP sheet surface using 25 μm-thick polyimide adhesive tape. A temperature sensor is attached to position *A* on the compression area of the molded product and position *B* on the rib part, which is located on the back surface of the CFRTP sheet and face core insert in the movable mold side, as shown in Figure 3b.

The surface of the CFRTP sheet is observed using a digital camera and a shape analysis laser microscope (VK-X160; Keyence Corporation, Osaka, Japan) to evaluate the quality of the appearance of the molded products. The observation areas are the upper part *C* and the compression area of the molded product *D* of the molded product, as shown in Figure 3b. The reason for selecting *C* and *D* as observation areas is that, after viewing the molded products sampled in this study, surface defects occur more frequently in the compression area at the end of the molding process.

## 3. Results

### 3.1. Carbon Fiber Tow Orientation

**Figure 10:**
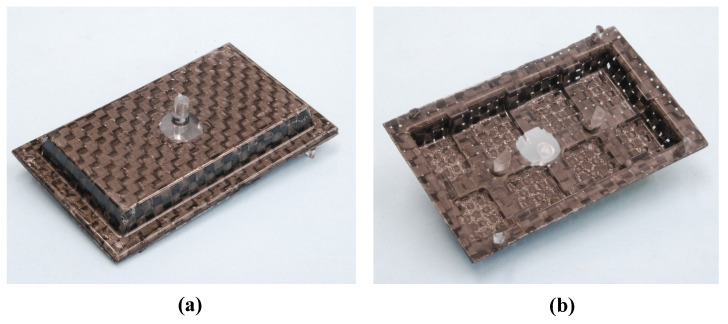
Appearance of the hybrid injection-molded product (one layer, *α* = 12.5%, 50 °C→280 °C→50 °C, Δ*t* = 10 s): (**a**) front; (**b**) back.

Figure 10 shows the appearance of the hybrid injection-molded product obtained using the CFRTP sheet with the fiber tow orientation of 0°/90°. The molded product is obtained under the following conditions: the number of sheet layers is one, *α* is 12.5%, the heating temperature is 50 °C → 280 °C → 50 °C, and Δ*t* is 10 s. By using the developed mold and a far-infrared radiation heater, it is confirmed that heat-and-cool hybrid injection-molded products can be obtained using only an injection molding machine. Using this molding technique, the effects of the sheet conditions and injection molding process conditions on molded products are examined, as described below.

**Figure 11:**
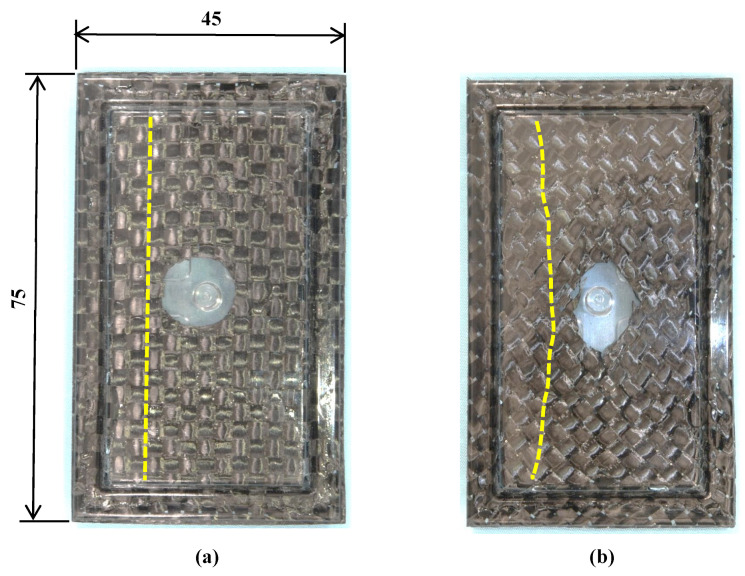
Molded products obtained by changing fiber tow orientation (one layer, *α* = 12.5%, 50 °C → 240 °C → 50 °C, Δ*t* = 15 s, Yellow dotted line: Line connecting the points where the fiber tows in two directions intersect.): (**a**) 0°/90°; (**b**) +45°/−45°.

Figure 11 shows the molded products obtained by changing the fiber tow orientation. The number of CFRTP sheet layers is one, *α* is 12.5%, the heating condition is 50 °C → 240 °C → 50 °C, and Δ*t* is 15 s. To examine the distortion of the woven pattern, the points where the fiber tows in two directions intersect are connected by yellow dotted lines. The position of the gate hole is displaced laterally from the center and the shape of the hole is deformed into an ellipse in both molded products. This is assumed to be because the CFRTP sheet moves slightly during compression molding, owing to the imbalanced clamping force of the sheet-holding plates. It has been observed that, if the clamping force is too large, the CFRTP sheet is not drawn into the cavity during compression molding, resulting in sheet fracture. These results indicate that caution is needed in adjusting the clamping force applied to the CFRTP sheet. Distortion occurs in the molded product with a +45°/−45° fiber orientation, whereas no distortion occurs in the molded product with a 0°/90° fiber orientation. This is assumed to be because in the 0°/90°-sheet-molded product, the orientation of the fiber tow is aligned with the direction in which the CFRTP sheet is pulled during compression molding. The subsequent experiments are conducted under the fiber tow orientation condition of 0°/90°, under which no distortion occurs.

### 3.2. Effects of Heating Temperature of Three-Dimensional Cavity Plate and Δt

**Figure 12:**
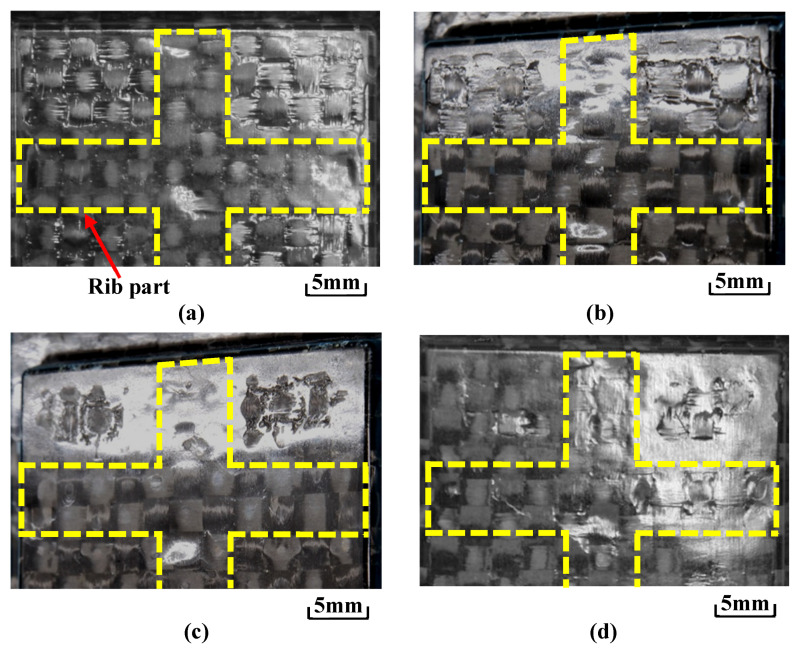
Digital camera images of observation area *C* (from Figure 3b) for molded products obtained by setting Δ*t* to 0 s and changing the heating temperature of the three-dimensional cavity plate (one layer, *α* = 12.5%): (**a**) 50 °C → 180 °C → 50 °C; (**b**) 50 °C → 200 °C → 50 °C; (**c**) 50 °C → 240 °C → 50 °C; (**d**) 50 °C → 280 °C → 50 °C.

**Figure 13:**
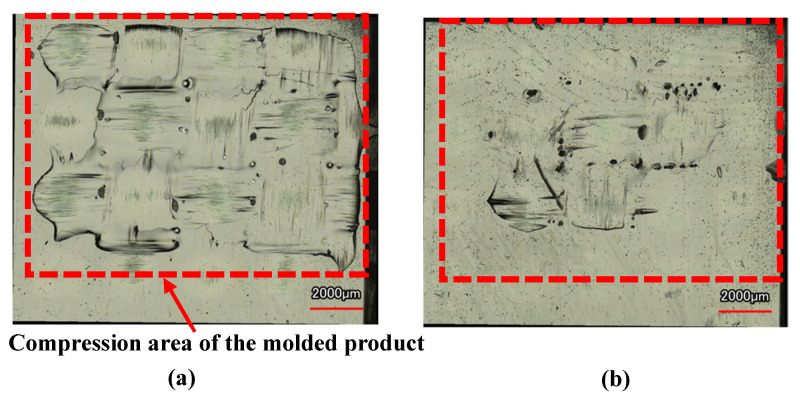
Shape analysis laser microscopy images of hybrid injection-molded product in area *D* in Figure 3b at Δ*t* = 0 s (one layer, *α* = 12.5%): (**a**) 50 °C → 180 °C → 50 °C; (**b**) 50 °C → 280 °C → 50 °C.

Figure 12 shows the digital camera images of observation area *C* (denoted in Figure 3b) for the molded products, obtained by setting Δ*t* to 0 s and changing the heating temperature of the three-dimensional cavity plate. The number of CFRTP sheet layers is one and *α* is 12.5%. The positions of the ribs molded on the back surface of the molded products are indicated by dotted lines. Dent surface defects are generated in the compression area of the molded products under all heating conditions. The surface defects do not disappear, even when the heating condition is 50 °C → 280 °C → 50 °C, although the surfaces of the molded products gradually become smooth with the increasing heating temperature. To observe the surface defects in detail, a shape analysis laser microscope is used to magnify the compression area *D* (denoted in Figure 3b), and the results are shown in Figure 13. The results at 50 °C → 180 → 50 °C and 50 °C → 280 → 50 °C are shown. Dent surface defects are generated in the compression area of the molded product under heating conditions of 50 °C → 180 °C → 50 °C. The fiber weave pattern is exposed at the bottom of the dents, which hereafter is referred to as the “exposed fiber weave pattern”. The surface defects do not completely disappear although the area where the surface defects are generated decreases with the increasing heating temperature.

**Figure 14:**
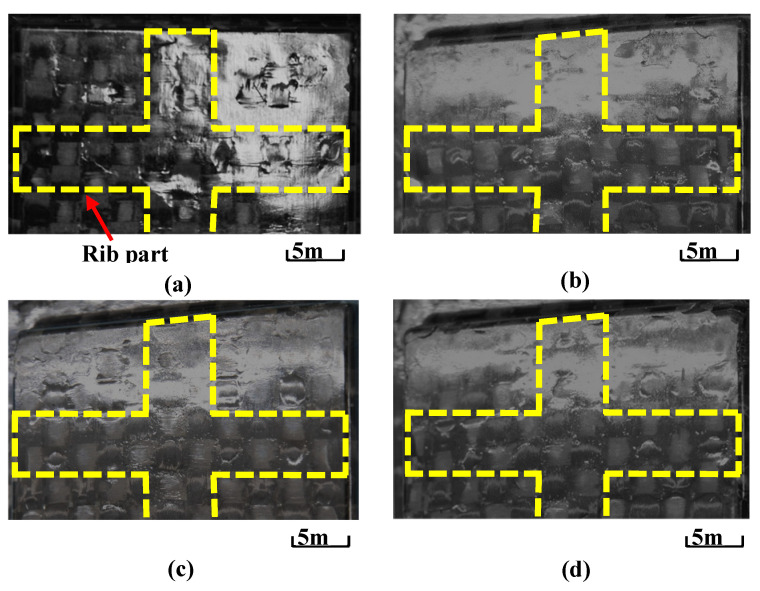
Digital camera images of observation area C (from Figure 3b) for molded products under heating conditions of 50 °C → 280 °C → 50 °C while changing Δ*t* (one layer, *α* = 12.5%): (**a**) Δ*t* = 0 s; (**b**) Δ*t* = 5 s; (**c**) Δ*t* = 10 s; (**d**) Δ*t* = 15 s.

**Figure 15:**
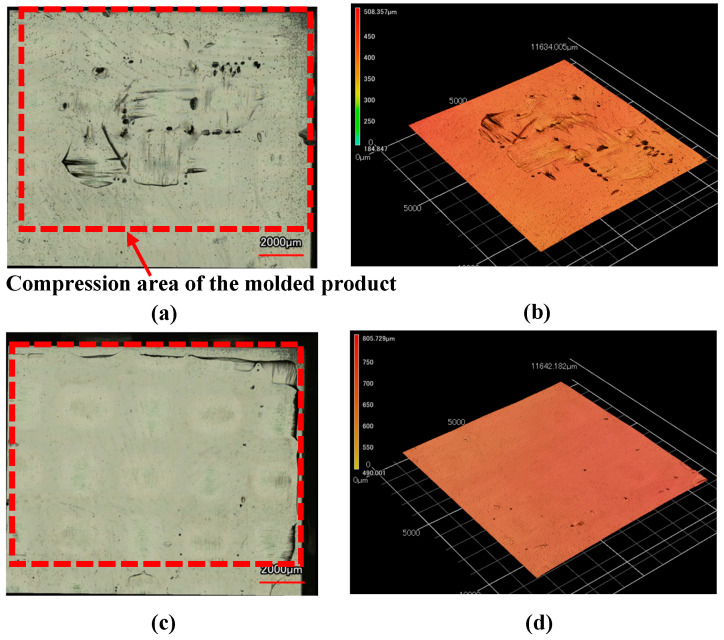
Shape analysis laser microscopy images and surface shape of hybrid injection-molded product in area *D* (from Figure 3b) for heating conditions of 50 °C → 280 °C → 50 °C (one layer, *α* = 12.5%): (**a**) Δ*t* = 0 s (laser microscopy image); (**b**) Δ*t* = 0 s (surface shape); (**c**) Δ*t* = 15 s (laser microscopy image); (**d**) Δ*t* = 15 s (surface shape).

Figure 14 shows the observation results of molded products formed by changing Δ*t* under the heating temperature conditions of 50 °C → 280 °C → 50 °C where surface defects do not completely disappear, as shown in Figure 13. The area where the exposed fiber weave pattern is generated gradually decreases with increasing Δ*t*. Figure 15 shows the images taken with a shape analysis laser microscope and the surface shapes of the molded products obtained by setting Δ*t* to 0 s and 15 s. The surface becomes smooth and the dents and the exposed fiber weave pattern completely disappear when Δ*t* is 15 s. A similar tendency is also observed under other heating conditions.

As described above, surface defects are generated when the heating temperature of the three-dimensional cavity plate is low. These defects do not completely disappear although the area where the surface defects are generated gradually decreases with the increasing heating temperature. Δ*t* strongly affects the appearance of molded products. Surface defects are generated in the compression area of the molded product when the melt is injected immediately after the completion of the compression molding of the CFRTP sheet. On the other hand, it is found that as Δ*t* increases, a flat surface profile without surface defects is generated on the molded product’s surface.

### 3.3. Effects of Number of CFRTP Sheet Layers and Compression Ratio

**Figure 16:**
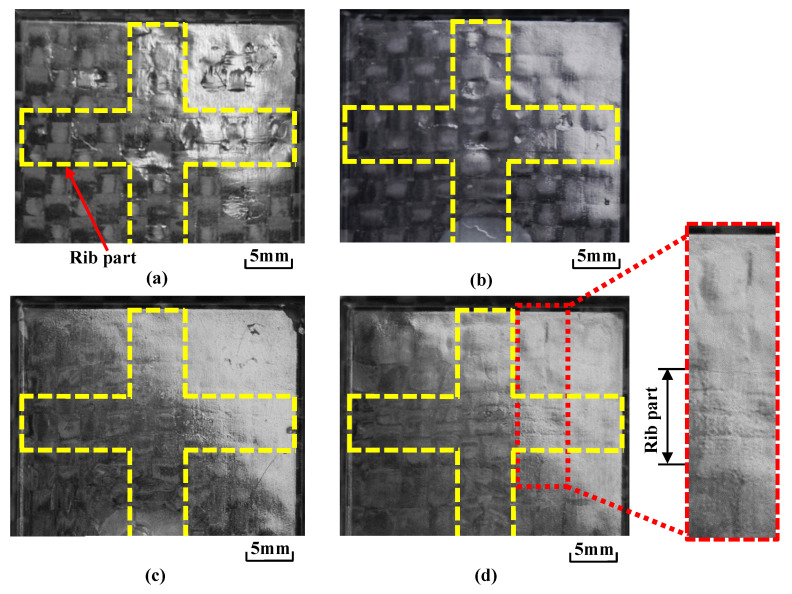
Digital camera images of observation area C (from Figure 3b) for molded products obtained by changing the number of CFRTP sheet layers and *α* at Δ*t* = 0 s for heating conditions of 50 °C → 280 °C → 50 °C: (**a**) one layer (*α* = 12.5%); (**b**) one layer (*α* = 37.5%); (**c**) two layers (*α* = 12.5%); (**d**) two layers (*α* = 37.5%).

**Figure 17:**
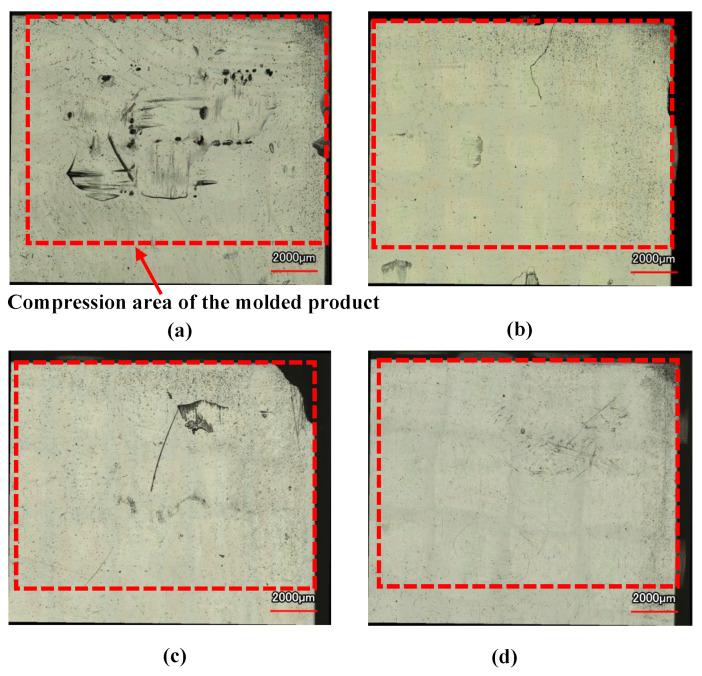
Shape analysis laser microscopy images of hybrid injection-molded product in area *D* in Figure 3b at Δ*t* = 0 s for heating conditions of 50 °C → 280 °C → 50 °C: (**a**) one layer (*α* = 12.5%); (**b**) one layer (*α* = 37.5%); (**c**) two layers (*α* = 12.5%); (**d**) two layers (*α* = 37.5%).

Figure 16 shows the molded products obtained by changing the number of CFRTP sheet layers and *α*. Figure 17 shows an enlarged view of the compression area of the molded product taken with a shape analysis laser microscope. The heating condition is 50 °C → 280 °C → 50 °C and Δ*t* is 0 s. When the number of CFRTP sheet layers is the same, the exposed fiber weave pattern is generated at a low *α* of 12.5%. The surface defects are not generated at a high *α* of 37.5%. When *α* is the same, the generation of the surface defects is suppressed more with two CFRTP sheet layers than with one CFRTP sheet layer, resulting in a glossy molded product surface. Molded product surfaces with excellent glossiness are generated when the number of CFRTP sheet layers is two and *α* is 37.5%. However, the fiber tow width in the compression area of the molded product is different from that in the rib part, resulting in an irregular weave pattern. In particular, the weave pattern in the rib part is distorted and appears to be compressed from the top and bottom, as shown in Figure 16d.

As described above, as the number of CFRTP sheet layers and the sheet compression ratio *α* increase, smooth molded product surfaces without defects are generated. However, the fiber weave pattern becomes irregular when the number of CFRTP sheet layers is two and the sheet compression ratio *α* is high.

### 3.4. Observation of Cross Sections of Molded Products

**Figure 18:**
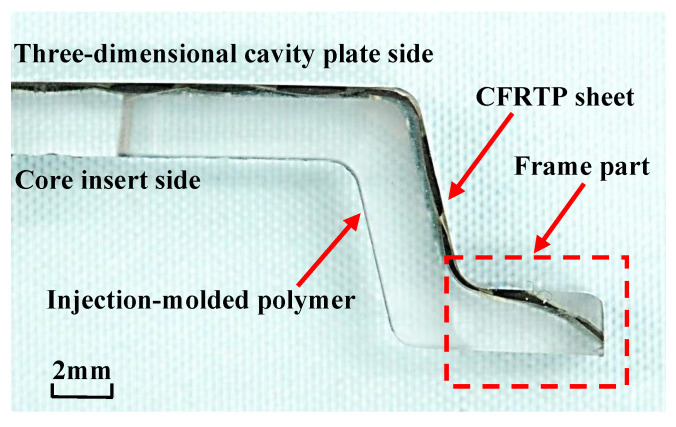
Cross section of hybrid injection-molded product at Δ*t* = 0 s for heating conditions of 50 °C → 280 °C → 50 °C (one layer, *α* = 12.5%, cross section *E* from Figure 3b).

Figure 18 shows the cross section *E* of the molded product (as denoted in Figure 3b). Both the front and back of the CFRTP sheet are embedded in the injection-molded polymer in the frame part of the molded product. This is assumed to be because the CFRTP sheet in the frame part is not sandwiched between the three-dimensional cavity plate and the core insert and is held aloft in the air during compression molding.

**Figure 19:**
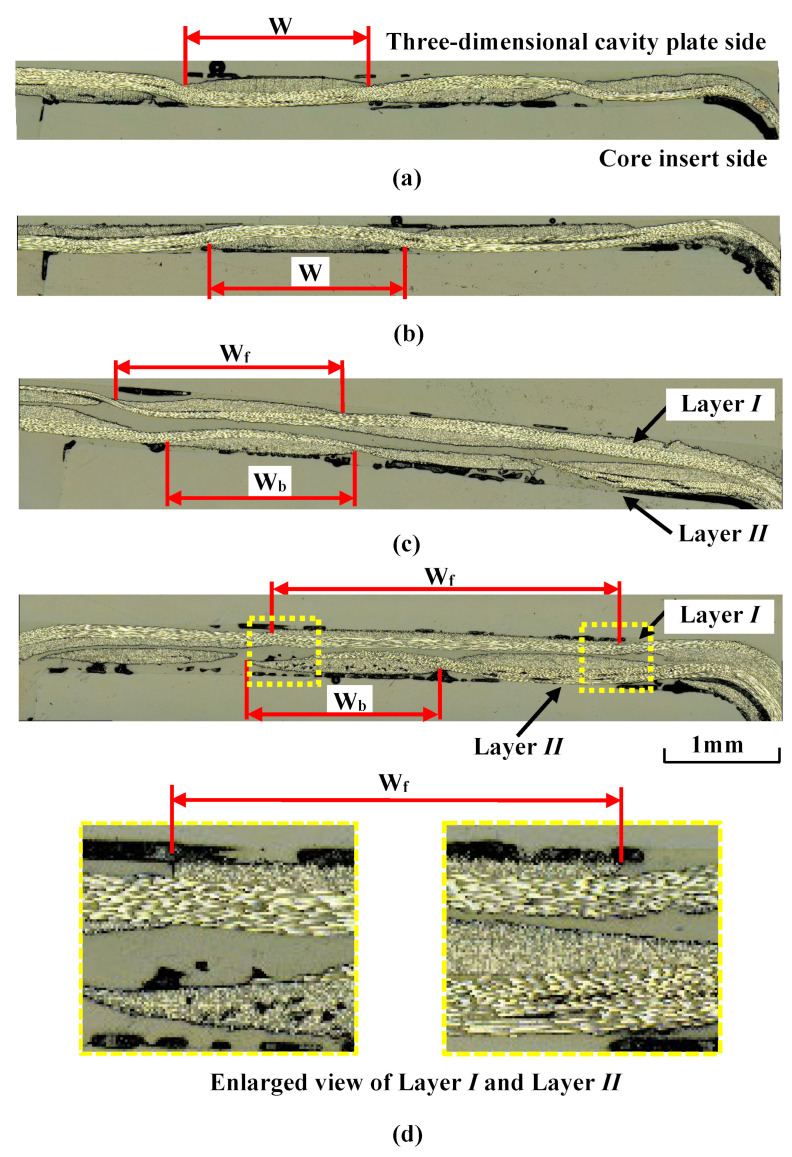
Cross section of fiber tow in the compression area of the molded product at Δ*t* = 0 s for heating conditions of 50 °C → 280 °C → 50 °C (cross section *F*-*F* in Figure 3b): (**a**) one layer (*α* = 12.5%); (**b**) one layer (*α* = 37.5%); (**c**) two layers (*α* = 12.5%); (**d**) two layers (*α* = 37.5%).

Figure 19 shows the cross section of the fiber tow in the compression area of the molded product obtained at different compression ratio *α* values when the numbers of CFRTP sheet layers are one and two. The heating condition is 50 °C → 280 °C → 50 °C and Δ*t* is 0 s. The width of the fiber tow, *W*, does not change with *α* in the molded products with one CFRTP sheet layer, as shown in Figure 19a,b. The width of the fiber tow in layer *II* on the core insert side, *W_b_*, changes little with *α* in the molded products with two CFRTP sheet layers, as shown in Figure 19c,d. However, the width of the fiber tow in layer *I* on the three-dimensional cavity plate side, *W_f_*, increases with *α* because the fiber tow is deformed in the thickness direction of the molded product and flattened. As a result, *W_f_* becomes larger than *W_b_*.

### 3.5. Results of Measurement of Sheet Temperature

**Figure 20:**
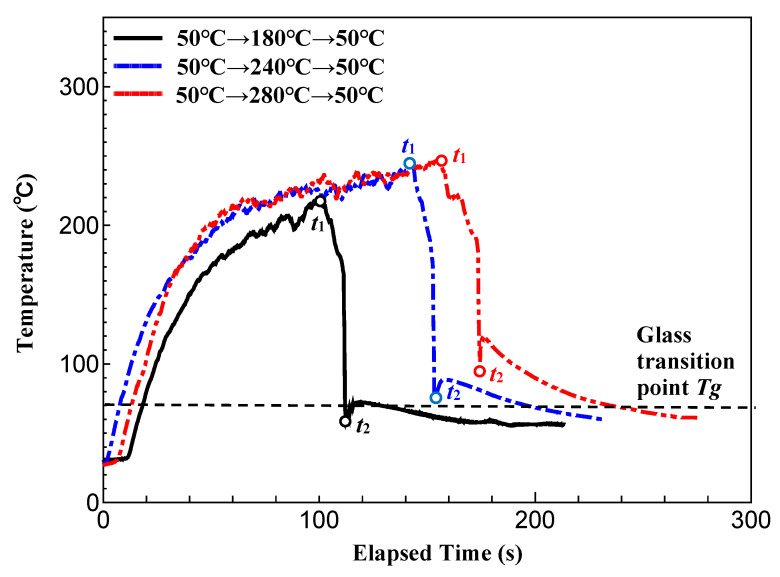
Temperature measurement results at position *A* in the CFRTP sheet obtained by changing the heating temperature of the three-dimensional cavity plate (one layer, *α* = 12.5%, Δ*t* = 0 s).

Figure 20 shows the temperature measurement results at position *A* in the CFRTP sheet when Δ*t* is 0 s, and the heating condition of the three-dimensional cavity plate is changed. The parameter *t*_1_ indicates the time to start lifting the far-infrared radiation heater from the three-dimensional cavity plate surface, and *t*_2_ indicates the time to complete compression molding. As the heating temperature of the three-dimensional cavity plate increases, the temperature of the CFRTP sheet increases during the time period from the start of the lifting of the far-infrared radiation heater (*t*_1_) to the completion of compression molding (*t*_2_).

At the time of *t*_1_, when the temperature of the three-dimensional cavity plate reaches its maximum, the surface temperature of the sheet is 210 °C, which is higher than the temperature of the plate under the condition of 50 °C → 180 °C → 50 °C. At the same time, for the condition of 50 °C → 280 °C → 50 °C, the surface temperature of the sheet is 240 °C, which is lower than the temperature of the plate. The causes of this are discussed below. The CFRTP sheet is hidden behind the three-dimensional cavity plate; therefore, it is difficult to heat it by radiation. At the start of heating, the surface temperature of the sheet, which has a smaller heat capacity than the plate, rises more rapidly. On the other hand, when it reaches a high temperature, the temperature of the plate reaches 280 °C by radiation and heat transfer from the heater, but that of the sheet does not reach 280 °C because heating by radiation is difficult.

The CFRTP sheet temperatures at *t*_1_ and *t*_2_ increase as the heating temperature of the plate increases. It is assumed that the viscosity of the base polymer of the CFRTP sheet decreases as the sheet temperature increases with the heating temperature [39], and the CFRTP sheet is more susceptible to molecular diffusion under the condition of a high temperature at the time of compression completion *t*_2_, resulting in the squeeze flow of the low-viscosity base polymer over the surface of the CFRTP sheet during compression molding. As a result, it is assumed that the surface properties of the three-dimensional cavity plate are easily transferred to the surface of the CFRTP sheet.

**Figure 21:**
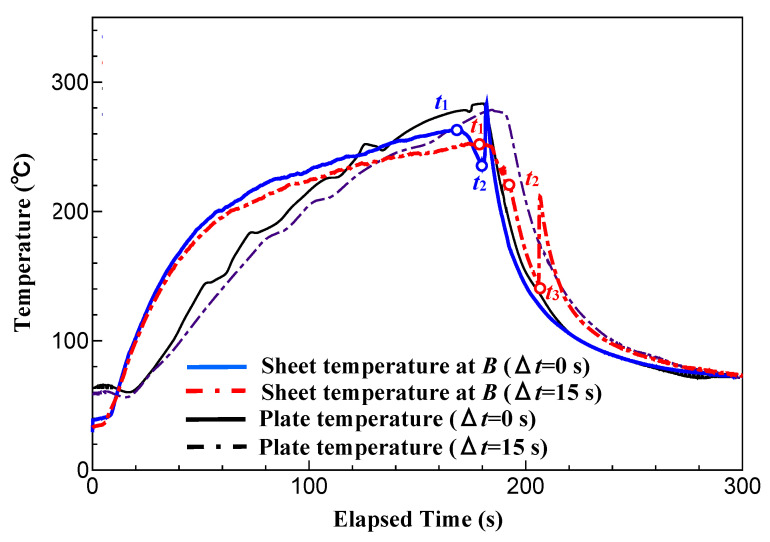
Temperature measurement results at position *B* in the rib part of CFRTP sheet and three-dimensional cavity plate during hybrid injection molding for heating conditions of 50 °C → 280 °C → 50 °C (one layer, *α* = 12.5%).

Figure 21 shows the temperature measurement results at position B in the rib part of the CFRTP sheet and the three-dimensional cavity plate during heat-and-cool hybrid injection molding, as measured while changing Δ*t*. When Δ*t* is 0 s, the sheet temperature decreases until the completion of compression molding (*t*_2_), then increases again, and decreases rapidly. On the other hand, when Δ*t* is 15 s, the sheet temperature decreases rapidly even after the completion of compression molding (*t*_2_), decreasing to 150 °C after 15 s (*t*_3_). The results suggest that the sheet temperature at position *A* in the compression area of the molded product, where both sides of the CFRTP sheet are in contact with the cavity surface, is lower than that at the completion of compression molding *t*_2_ in Figure 20. The sheet temperature again increases when the melt is injected 15 s later. This is a result of the two-stage molding process: (1) the compression molding stage—heat is transferred from the CFRTP sheet to the cavity surface when they come into contact, causing a decrease in the sheet temperature; (2) injection molding stage—heat is transferred from the injection-molded polymer to the CFRTP sheet, causing an increase in the sheet temperature. However, the sheet temperature when the melt is injected 15 s later is lower than that at the completion of compression molding (*t*_2_). Under such temperature condition, it is difficult for molecules to diffuse at the interface between the base polymer of the CFRTP sheet and the injection-molded polymer, and the adhesive strength between the sheet and the polymer is presumably lower than in the case of Δ*t* = 0 s.

As described above, it is found that the heating temperature of the three-dimensional cavity plate and Δ*t* affect the temperature of each part of the CFRTP sheet during injection molding.

## 4. Discussion

Surface defects with the exposed fiber weave pattern are generated in the compression area of the molded product when Δ*t* is 0 s, namely, when injection molding is started immediately after the compression molding of the CFRTP sheet. On the other hand, molded products having a smoother surface without defects are obtained when Δ*t* is increased. The sheet temperature increases with the heating temperature of the three-dimensional cavity plate. The viscosity of PMMA, the base polymer of the CFRTP sheet, decreases in the compression area of the molded product during compression molding. As a result, the base polymer squeezes out onto the surface of the CFRTP sheet and adheres to the three-dimensional cavity plate. This is assumed to be one possible reason that the surface properties of the three-dimensional cavity plate are fully transferred to the molded product’s surface, resulting in mold products with excellent surface properties. However, molded products with the optimal surface properties are not obtained when only the heating temperature is increased. It is found that the surface defects completely disappear only when Δ*t* is increased. Moreover, surface defects are less likely to be generated when the number of CFRTP sheet layers and the sheet compression ratio *α* are high. These results are discussed below.

**Figure 22:**
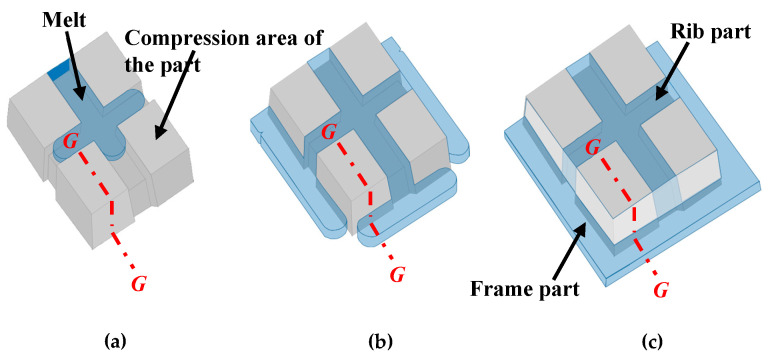
Diagrams of the predicted behavior of melt flowing from the rib part to the frame part of the mold: (**a**) melt flowing process inside the rib part; (**b**) melt flowing process inside the frame part; (**c**) melt full packing process.

Figure 22 shows a diagram of the predicted behavior of the melt flowing from the rib part to the frame part of the mold. Only the runner and the melt in the frame part are drawn while the CFRTP sheet is omitted in this figure. The melt passes through the rib part, as shown in Figure 22a, and flows into the frame part, as shown in Figure 22b. The frame part becomes fully filled with the melt when the melt fronts meet at the corners of the frame part, as shown in Figure 22c.

**Figure 23:**
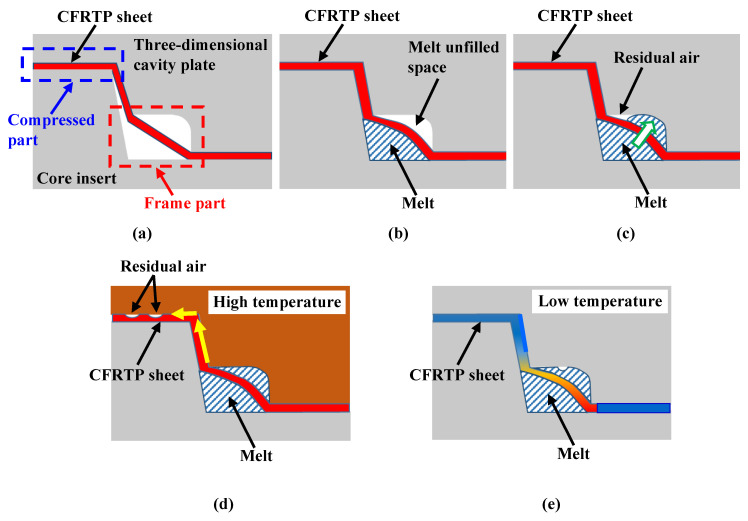
Diagram of the estimated mechanism of generation of surface defects with exposed fiber weave pattern in the compression area of the molded product (cross section *G*–*G* in Figure 22c): (**a**) immediately after compression molding; (**b**) melt flowing process inside the frame part on the core insert side; (**c**) melt flowing process inside the frame part on the three-dimensional plate side; (**d**) melt full packing process under high-temperature conditions; (**e**) melt full packing process under low-temperature conditions.

Figure 23 shows a diagram of the estimated mechanism of the generation of surface defects with the exposed fiber weave pattern at the *G–G* cross section in Figure 22c. Immediately after compression molding, as shown in Figure 23a, the sheet remains softened inside the frame, without being cooled, because the sheet is not sandwiched between the three-dimensional cavity plate and the core insert. Then, the melt flows through the space on the core insert side of the sheet in the frame part when injection starts, as shown in Figure 23b. The melt pressure increases after the space on the core insert side of the sheet is filled with the melt. As a result, the melt flows through the gaps between the fiber tow of the sheet into the unfilled space on the three-dimensional cavity plate in the frame part, as shown in Figure 23c. In this study, the pressure distributed on the CFRTP sheet and the melt pressure are not measured by the pressure sensor. Therefore, the following discussion is only speculative. Assuming that the holding pressure of 60 MPa is directly applied to the melt inside the frame part if ignoring the pressure loss, it can be considered that the pressure distributed in the sheet of the compression area of the molded product is lower than the pressure of the melt inside the frame area under the low *α* condition. As a result, the residual air in the unfilled space is pushed by the melt toward the contact area between the CFRTP sheet and the three-dimensional cavity plate, and it moves into the compression area of the molded product, as shown in Figure 23d. When Δ*t* is short, the viscosity of PMMA, the base polymer of the CFRTP sheet, is low because PMMA is maintained at a high temperature. It is assumed that the air moving into the compression area of the molded product pushes the PMMA inward in the compression area of the molded product, resulting in the generation of dent surface defects with the exposed fiber weave pattern. When Δ*t* is long, the residual air is also pushed to the compression area of the molded product. However, as shown in Figure 23e, the air diffuses throughout the contact area between the CFRTP and the three-dimensional cavity plate because the sheet in the compression area of the molded product has already cooled and solidified as the heat from the sheet is transferred to the cavity wall. As a result, it is assumed that the above surface defects are not generated. When *α* is high, it is assumed that little air penetrates the contact area between the CFRTP sheet and the three-dimensional cavity plate because the pressure inside PMMA in the compression area of the molded product is higher than the melt inside the frame part due to stronger compression. The surface defects are, therefore, not generated.

**Figure 24:**
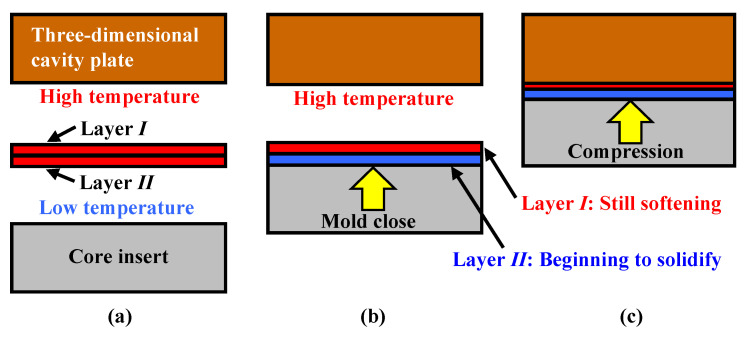
Diagram of estimated compression behavior of CFRTP sheets during compression molding: (**a**) before compression; (**b**) mold-closing process; (**c**) mold-clamping process.

Figure 24 demonstrates why surface defects are not generated when the number of CFRTP sheet layers is two, even when *α* is the same. As seen in the cross section in the compression area of the molded product shown in Figure 19d, only the fiber tow of layer *I* on the three-dimensional cavity plate side is greatly deformed when the number of CFRTP sheet layers is two and *α* is 37.5%. When the CFRTP sheet has two layers, layer *II* of the CFRTP sheet is on the core insert side, as shown in Figure 24a. When mold clamping starts, layer *II* first comes into contact with the core insert, which is kept at a low temperature and begins to solidify from this moment, as shown in Figure 24b. The thickness of layer *II*, whose viscosity has increased since solidification began, changes little even when it is further compressed during mold clamping. On the other hand, the thickness of layer *I*, which is still softened, largely decreases when it is compressed. Layer *I* is significantly deformed, resulting in increased pressure inside the PMMA. Therefore, little air penetrates the contact area between the CFRTP sheet and the three-dimensional cavity plate. This is assumed to be the reason that surface defects are not generated when the CFRTP sheet has two layers. It is also assumed that as *α* increases, layer *I* is greatly deformed in the compression area of the molded product, protruding out of the compression area of the molded product and into the rib part, compressing the CFRTP sheet in the rib part. As a result, the weave pattern becomes irregular in the rib part, as shown in Figure 16d.

## 5. Conclusions

The following conclusions are obtained from this study:

1. A mold and a molding technique using a far-infrared radiation heater are proposed to enable a simple experimental analysis of the effects of the mold heating temperature and injection molding process conditions on the appearance quality of molded products in the heat-and-cool hybrid injection molding of CFRTP sheets. It is then demonstrated that heat-and-cool hybrid injection-molded products can be obtained using this molding technique.
2. The elapsed time Δ*t* between the completion of the compression molding of the CFRTP sheet and the start of melt injection strongly affects the appearance of the molded products. Surface defects with the exposed fiber weave pattern are generated in the compression area of the molded product when Δ*t* is short. On the other hand, smoother molded product surfaces without defects are generated when Δ*t* is increased.
3. Surface defects in the molded products cannot be suppressed simply by increasing the temperature of the three-dimensional cavity plate. In addition, the above defects can be suppressed by lengthening Δ*t*.
4. The compression ratio *α* of the CFRTP sheet strongly affects the appearance of the molded products. Smoother molded product surfaces without defects are generated when *α* is increased.
5. When the CFRTP sheet has two layers, there is a difference in the solidification conditions between the two layers. As a result, the weave pattern becomes irregular although the surface defects with the exposed fiber weave pattern that are generated in one layer of the CFRTP sheet are not generated.

As described above, the effectiveness of the proposed heat-and-cool hybrid injection mold and the molding technique using this mold for CFRTP sheets is demonstrated through the experimental analysis of surface defects. Only the CFRTP sheet with the PMMA base polymer, a general-purpose polymer, is examined in this study. CFRTP sheets based on engineering plastics are widely used in industrial fields. In the future, further improvements in the heating characteristics of the three-dimensional cavity plate and CFRTP sheet are expected so that they can be applied to the experimental analysis of molding phenomena in the above sheets.

## Figures and Tables

**Table 1:** Molding process conditions.

Cylinder Temperature	(°C)	260–260–250–210–50 *
Hot sprue temperature	(°C)	260
Mold base temperature	(°C)	50
Injection rate	(cm^3^/s)	21.3
Holding pressure	(MPa)	60
Holding pressure period	(s)	15
Compression force	(kN)	500
Far-infrared radiation heater temperature	(°C)	600
Three-dimensional plate temperature	(°C)	(1) 50 → 180 → 50 (2) 50 → 200 → 50
		(3) 50 → 240 → 50 (4) 50 → 280 → 50
Elapsed time between completion of compression molding of CFRTP sheet and start of melt injection Δ*t*	(s)	0, 5, 10, 15
Carbon fiber tow orientation angle	(deg)	0/+90, +45/−45
Number of CFRTP sheet layers	(layer)	1, 2
Compression ratio of CFRTP sheets *α*	(%)	12.5, 37.5

* Nozzle–Metering–Compression–Feed Zone–Under Hopper.

## Data Availability

The data that support the findings of this study are available on request from the corresponding author.
